# Integration of Metagenomic and Stable Carbon Isotope Evidence Reveals the Extent and Mechanisms of Carbon Dioxide Fixation in High-Temperature Microbial Communities

**DOI:** 10.3389/fmicb.2017.00088

**Published:** 2017-02-03

**Authors:** Ryan de Montmollin Jennings, James J. Moran, Zackary J. Jay, Jacob P. Beam, Laura M. Whitmore, Mark A. Kozubal, Helen W. Kreuzer, William P. Inskeep

**Affiliations:** ^1^Department of Land Resources and Environmental Sciences, Montana State UniversityBozeman, MT, USA; ^2^Thermal Biology Institute, Montana State UniversityBozeman, MT, USA; ^3^Pacific Northwest National LaboratoriesRichland, WA, USA

**Keywords:** autotrophy, CO_2_ fixation, stable C isotopes, geothermal, Aquificales, Crenarchaeota

## Abstract

Although the biological fixation of CO_2_ by chemolithoautotrophs provides a diverse suite of organic compounds utilized by chemoorganoheterotrophs as a carbon and energy source, the relative amounts of autotrophic C in chemotrophic microbial communities are not well-established. The extent and mechanisms of CO_2_ fixation were evaluated across a comprehensive set of high-temperature, chemotrophic microbial communities in Yellowstone National Park by combining metagenomic and stable ^13^C isotope analyses. Fifteen geothermal sites representing three distinct habitat types (iron-oxide mats, anoxic sulfur sediments, and filamentous “streamer” communities) were investigated. Genes of the 3-hydroxypropionate/4-hydroxybutyrate, dicarboxylate/4-hydroxybutyrate, and reverse tricarboxylic acid CO_2_ fixation pathways were identified in assembled genome sequence corresponding to the predominant Crenarchaeota and Aquificales observed across this habitat range. Stable ^13^C analyses of dissolved inorganic and organic C (DIC, DOC), and possible landscape C sources were used to interpret the ^13^C content of microbial community samples. Isotope mixing models showed that the minimum fractions of autotrophic C in microbial biomass were >50% in the majority of communities analyzed. The significance of CO_2_ as a C source in these communities provides a foundation for understanding community assembly and succession, and metabolic linkages among early-branching thermophilic autotrophs and heterotrophs.

## Introduction

Despite the significance of microorganisms in nearly all major element cycles, the contribution of microbial inorganic carbon (C) fixation to global C cycling is not yet well-resolved (Shively et al., 2001; Reinthaler et al., 2010). Resolution of the C cycle is important across multiple scales, ranging from specific microbial communities to regional environments, and ultimately, to the global Earth system. On an energetic basis, the reduction of CO_2_ to biomass C is extremely costly and organisms obtain this energy either from photons (i.e., phototrophy) or the oxidation of reduced chemical species (i.e., chemotrophy). The plethora of inorganic electron donors that are available to drive chemolithoautotrophy include reduced forms of hydrogen, sulfur, nitrogen, arsenic, carbon, iron, and manganese. The fixation of CO_2_ occurs via one of six currently known biochemical pathways in *Archaea, Bacteria*, and/or *Eukarya*. Therefore, resolution of C cycling at the microbial community scale requires the integration of metabolic and molecular data (e.g., metagenome sequence) with geochemical studies, such as stable carbon isotope (^13^C) analysis or isotope probing. Stable isotope probing (SIP) using ^13^CO_2_ and subsequent analysis of heavy DNA allows for the identification of organisms responsible for the fixation of CO_2_ in microbial communities; however, SIP is technically challenging *in situ* across a large number of different environments. Moreover, other measurements are required to gain appreciation for the relative extent of autotrophy vs. heterotrophy in mixed microbial communities. Direct analysis of stable carbon isotopes (i.e., ^13^C) has been employed to dissect the trophic structure of numerous different ecosystems, and can provide primary evidence for the sources of C to specific biota (Peterson and Fry, 1987).

Currently established pathways responsible for the fixation of CO_2_ in chemotrophic organisms that are known inhabitants of iron-oxide, sulfur-sediment, and filamentous “streamer” communities in Yellowstone National Park (YNP, Wyoming, USA) include the reductive tricarboxylic acid cycle, reductive acetyl-CoA pathway, 3-hydroxypropionate/4-hydroxybutyrate cycle, and the dicarboxylate/4-hydroxybutyrate cycle (Berg et al., 2010; Hügler and Sievert, 2011). The reverse tricarboxylic acid (r-TCA) cycle has been described in numerous lineages of *Bacteria*, including the hyperthermophilic Aquificales, which are known to inhabit marine hydrothermal vents and geothermal environments (Reysenbach et al., 2000, 2005; Huber and Eder, 2006). The reductive acetyl-CoA (Wood-Ljungdahl) pathway has been described in acetogenic bacteria and methanogenic archaea, as well as sulfate-reducing bacteria (Hügler and Sievert, 2011), which are found in hypoxic environments including sediments, soils, sludge, and intestinal tracts (Breznak and Kane, 1990; Whitman et al., 2006; Drake et al., 2008). The 3-hydroxypropionate/4-hydroxybutyrate (3-HP/4-HB) cycle has been identified in several major lineages of *Archaea*, including the Crenarchaeota (order Sulfolobales) and marine Thaumarchaeota (Berg et al., 2007; Walker et al., 2010; Könneke et al., 2014). The dicarboxylate/4-hydroxybutyrate (DC/4-HB) cycle utilizes a PEP carboxylase and has been identified in the Thermoproteales and Desulfurococcales (phylum Crenarchaeota; Huber et al., 2008; Ramos-Vera et al., 2009); the regenerative half of this cycle (i.e., succinyl-CoA to acetyl-CoA) is identical to the 3-HP/4-HB cycle.

Energy-rich hydrothermal and geothermal environments contain deeply-branching thermophiles basal to the tree of life and are thought to be analogous to environments potentially common on early Earth (Nisbet and Sleep, 2001). Numerous high-temperature geothermal environments in Yellowstone National Park (YNP, WY, USA) have been characterized (e.g., Inskeep et al., 2013a) and provide opportunities for examining non-phototrophic CO_2_ fixation across a range of habitat types. Prior efforts to determine the importance of CO_2_ fixation in high-temperature microbial communities have included stable isotope measurements (Estep, 1984; Jahnke et al., 2001; Havig et al., 2011), ^14^CO_2_ incorporation assays (Boyd et al., 2009), and genome-enabled analysis of microbial communities and important isolates (Kozubal et al., 2008; Inskeep et al., 2010, 2013b; Takacs-Vesbach et al., 2013). However, there have been no systematic studies reporting on the ^13^C content of microbial community biomass present in non-phototrophic thermal environments. Consistent δ^13^C (‰) values of dissolved inorganic and organic C (i.e., DIC and DOC) have averaged approximately −3 and −23%, respectively, across different geothermal springs in YNP (Craig, 1953a,b; Bergfeld et al., 2014). We recently utilized the large difference in ^13^C content between inorganic and organic C pools to evaluate the extent of CO_2_ fixation in acidic Fe(III)-oxide microbial mats (Jennings et al., 2014), and showed that CO_2_ fixation contributed no less than ~40% of the microbial C in biomineralized Fe-oxide mat communities.

Here, we expand this approach to integrate random metagenome sequence analysis to identify the relative abundance of candidate autotrophs, and their respective CO_2_ fixation pathways, then utilize this information to evaluate the extent of CO_2_ fixation using stable ^13^C analysis of biomass C, dissolved inorganic C and organic C content of 15 different high-temperature microbial communities in YNP where phototrophic organisms are not important community members. The primary objectives of this study were to (i) determine the stable ^13^C isotope contents of dissolved inorganic and organic C, landscape organic C, and microbial mat or sediment C from major types of geothermal environments in YNP, (ii) identify the relative abundance of different chemotrophic microbial populations capable of CO_2_ fixation across sites, as well as the respective pathways potentially responsible for this metabolism, and (iii) evaluate the extent of microbial CO_2_ fixation in chemotrophic communities using isotope mixing models constructed with information from metagenome sequence data of these same sites. Results showed that CO_2_ fixation is an important metabolic process in nearly all high-temperature (T ~ 70–85°C) chemotrophic systems studied, which included sulfur-sediments, Fe(III)-oxide mats, and filamentous “streamer” communities ranging in pH from 2.5 to 9.

## Materials and methods

### Sample locations

The geothermal samples (Table 1, Table S1) obtained in the current study encompass a wide range of chemotrophic environments that capture major phylogenetic groups known to inhabit geothermal systems of YNP (Inskeep et al., 2010, 2013a,b; Kozubal et al., 2012; Takacs-Vesbach et al., 2013; Beam et al., 2016a,b; Jay et al., 2016). These sites have been the subject of extensive prior characterization and represent three distinct habitat types: hypoxic sulfur sediments, Fe(III)-oxide microbial mats, and filamentous “streamer” communities. The pH and temperature of these geothermal environments range from 2.0 to 9.4, and 68–87°C, respectively (Table 1).

**Table 1:** **Key geochemical parameters, and ^**13**^C stable isotope content (δ, ‰) of dissolved inorganic C, dissolved organic C (DIC, DOC) and microbial mat C^**1**^ determined across 15 different high-temperature springs in Yellowstone National Park (WY, USA)**.

**Site type**	**pH**	**T**	**DIC**	**DOC**	**δ^13^C DIC**	**δ^13^C DOC**	**δ^13^C Microbial Mat^a^**
**Location^b^**		**°C**	**mM**	**mM**	**(‰) (σ, *n*)**	**(‰) (σ, *n*)**	**(‰) (σ, *n*)^c^**
**SULFUR SEDIMENTS**							
Crater Hills	2	76	0.13	0.21	5.4 (0.2, 2)	−23.8 (0.2, 3)	−20.3 (0.7, 17)
Joseph's Coat 2 E	2.2	79	3.36	0.06	−5.4 (0.1, 2)	−23.9 (1.5, 2)	−16.1 (0.5, 7)
Monarch Geyser	3.8	83	1.15	0.05	−3.9 (0.8, 4)	−17.6 (1.8, 4)	−5.9 (1.1, 39)
Cistern Spring	4.5	79	0.67	0.04	−4.3 (0.6, 4)	−18.3 (1.5, 4)	−18.6 (3.6, 47)
Josephs Coat 3 A	6.1	87	0.38	0.05	−2.4 (0.2, 2)	−16.9 (0.2, 2)	−13.8 (1.0, 9)
Washburn Spring	6.2	72	3.85	1.01	0.6 (0.5, 2)	−22.3 (0.7, 2)	−23.4 (0.4, 9)
**ACIDIC FE-OXIDE MATS**							
Joseph's Coat 2 B	2.2	80	0.05	0.05	−3.4 (1.4, 4)	−23.7 (1.1, 5)	−8.3 (0.5, 7)
Beowulf Spring D	2.9	68	1.02	0.05	−3.2 (3.1, 8)	−22.7 (1.4, 8)	−15.4 (1.5, 41)
Grendel Spring D	3.4	70	0.2	0.06	−3.9 (0.6, 4)	−21.7 (0.6, 4)	−16.3 (0.7, 15)
Echinus Geyser B	3.5	68	0.26	0.03	−4.4 (0.4, 5)	−22.4 (1.0, 5)	−11.5 (0.8, 32)
100 Springs Plain B	3.5	74	0.23	0.06	−3.7 (2.9, 8)	−22.9 (2.3, 8)	−14.5 (1.6, 22)
**STREAMER COMMUNITIES**							
Dragon Spring B	3	75	1.62	0.05	−3.4 (0.3, 2)	−23.9 (0.9, 2)	−9.1 (0.8, 7)
Narrow Gauge	6.3	72	13.16	0.07	1.2 (1.2, 4)	−20.8 (3.5, 4)	−11.9 (0.6, 22)
Octopus Spring B	8.1	84	5.19	0.02	−1.9 (0.2, 7)	−23.0 (1.7, 7)	−16.3 (0.6, 13)
Conch Spring B	9.4	85	3.43	0.02	−2.5 (0.2, 9)	−21.8 (3.4, 9)	−4.5 (2.6, 9)

a *δ^13^C Microbial Mat = sulfur sediment, iron oxide mat, or filamentous “streamer” samples*.

b *Site locations (GPS coordinates) and more detailed information on sampling dates are provided in Table S1. Site photographs are provided in Figure S1*.

c *Standard deviation and n are based on total number of sample and machine replicates, and include samples obtained at different sampling dates, when appropriate (Table S1)*.

### Carbon stable isotope analysis

Water samples were collected on site, filtered (0.2 μm) into sterile Falcon tubes, and analyzed for isotope content (^13^C) and total concentration of dissolved inorganic and dissolved organic C at the Colorado Plateau Stable Isotope facility (Flagstaff, AZ). Solid phase sediment, mats, and/or streamer samples were obtained using a sterile spatula, placed into sterile 50 mL Falcon tubes, and frozen until analysis. Samples used to evaluate the isotopic composition of “landscape” carbon were obtained using the same protocols from surrounding soils, vegetation, animal dung, and algal mats. Numerous sites were sampled multiple times over 3 years (details for each geothermal location are provided in Table S1).

Stable carbon isotope ratios (δ^13^C) of mat biomass and landscape samples were measured using a Thermo-Finnegan (Bremen, Germany) Delta V Plus isotope ratio mass spectrometer coupled to a Costech Analytical Technologies (Valencia, CA, USA) 4010 Elemental Analyzer and a Zero-Blank autosampler. Approximately 8–15 mg of lyophilized sediment, mat, streamer, or landscape sample was placed into tin capsules (Costech Analytical Technologies) and analyzed. Samples containing calcium carbonate (i.e., Conch and Narrow Gauge) were pre-treated overnight in 1 M HCl to remove carbonate minerals. Data were corrected using the point-slope method (Coplen et al., 2006) to in-house Pacific Northwest National Laboratory (PNNL) glutamic acid standards, which were calibrated to U.S. Geological Survey (USGS) Standard 40 (δ^13^C = −26.39%) and USGS Standard 41 (δ^13^C = +37.63%) glutamic acid isotopic standards. Carbon stable isotope content is measured as a ratio, R (^13^C/^12^C), then reported as a delta (δ) value, where δ equals (R_SA_/R_Std_ − 1) × 1000 and R_SA_ and R_Std_ are the isotope ratios of the sample and the internationally recognized standard, PeeDee Belemnite (PDB).

### Random metagenome analysis of predominant phylotypes

Assembled genome sequences of numerous thermophilic microorganisms from YNP geothermal environments (primarily genus level) were constructed primarily from Sanger and/or pyro-sequencing (Kozubal et al., 2012, 2013; Inskeep et al., 2013a,b; Takacs-Vesbach et al., 2013; Jay et al., 2016). These metagenome sequence assemblies were annotated using protocols established at the DOE-JGI in the IMG/Expert Review system (Markowitz et al., 2012), and have been deposited with the Department of Energy Joint Genome Institute (DOE-JGI) Integrated Microbial Genomes/Metagenome site (IMG/M) under corresponding Genome ID values (see Table S2 for full list of phylotypes). These sequence assemblies were used as high-quality reference sequences to bin random Illumina metagenome sequences from many of the same sites, perform relative abundance calculations, and to assess the potential for CO_2_ fixation based on genes identified in known biochemical pathways. Results for the reverse TCA, 3-HP/4-HB and/or DC/4-HB cycles (Beh et al., 1993; Hügler et al., 2005; Berg et al., 2007; Ramos-Vera et al., 2009) are summarized in Supplemental Information (Tables S3, S4). Significant evidence of the Calvin and 3-HP cycles was not observed in the organisms and habitats studied, though forms of RubisCO can occasionally be identified in metagenomes of high-temperature systems. The lack of evidence for the Calvin and 3-HP cycles is consistent with the absence of phototrophic microorganisms (e.g., Cyanobacteria and Chloroflexi). Microbial populations potentially utilizing the reductive acetyl-CoA pathway (i.e., acetogens, methanogens, and sulfate-reducing bacteria) were not dominant members of these communities, and genes coding for marker proteins in these pathways were not observed consistently in the reference sequence assemblies obtained from these sites. Consequently, the Calvin and 3-HP cycles, and the reductive acetyl-CoA pathway were not highly-represented in the habitats studied and were not considered as possible mechanisms contributing to CO_2_ fixation.

### Relative abundance of different inorganic C fixing microorganisms

Additional microbial mat samples from the same sites were transported on dry ice, and stored at −80°C prior to DNA extraction, and subjected to random Illumina metagenome sequencing at the DOE-Joint Genome Institute (Community Sequencing Program 701) (Table S5). These samples were also subjected to 16S rRNA gene amplicon sequencing using Illumina barcoded samples (iTags). Random metagenome sequence reads from Illumina sequencing were compared to *de novo* genome assemblies (or isolate genomes) generated previously using Sanger and/or 454 pyrosequencing (Inskeep et al., 2013a,b; Takacs-Vesbach et al., 2013). Individual sequence reads were binned (at 90% nucleotide identity) to respective phylotype genome assemblies and the relative abundance (RA) of individual populations (genera level or higher) was estimated as the percent of sequence reads binned compared to the total sequences available. Although not all phylotypes across all sites have been curated, with the exception of Washburn Spring, ~70–90 percent of individual random sequences were assigned to well-characterized phylotypes at 90% nucleotide identity (Table S6). Results from deep 16S rRNA gene sequencing (Illumina iTags) were used for determining relative population abundances in two sites. In the these cases, operational taxonomic units (OTU) were established at 97% nucleotide identity for short fragment iTag sequences, and then identified at >90% nucleotide identity by comparison to an internal reference set of long-fragment (>1200 nt) 16S rRNA gene sequences from these same geothermal features in YNP. All long-fragment reference 16S rRNA gene sequences are available on NCBI.

### ^13^C mixing models

The ^13^C stable isotope contents of microbial mat samples were predicted using site-specific mixing models, formulated on δ^13^C values of dissolved inorganic C (DIC), dissolved organic C (DOC), and landscape organic C (OC), as well as the weighted fractionation factor based on the relative abundance of CO_2_-fixing microorganisms in the community. The measured δ^13^C of sulfur sediments, Fe(III)-oxide mats, and filamentous “streamer” communities may contain C from microbial biomass of DIC-origin, microbial biomass of OC-origin, and non-thermophilic biomass of landscape organic C. Given the range of observed δ^13^C-DIC values (−5.4 to 5.4%) and the statistically similar δ^13^C content of DOC and exogenous organic C (mean = −22.8%, σ = 3.0), ^13^C mixing models were developed for each system based on the measured δ^13^C-DIC, and the mean δ^13^C of dissolved organic carbon (DOC) and landscape C for all systems. The stable carbon isotope contents (^13^C) of all samples were predicted as a function of the ratio of microbial biomass of DIC-origin relative to total microbial biomass (f_Microbial-DIC_, where f_Microbial-DIC_ + f_Microbial-DOC/OC_ = 1), and the ratio of biomass C relative to total mat C (f_Microbial-C_, where f_Microbial-C_ + f_Non-microbial-C_ = 1) using the following relationships:

(1) $$\begin{array}{l} \delta^{13}C_{\text{sample}}=\left(\delta^{13}C_{\text{biomass}}\times f_{\text{Microbial-C}}\right)\\ +\;\left(\delta^{13}C-OC\times f_{\text{Non-microbial-C}}\right) \end{array}$$

(2) $$\begin{array}{l} \delta^{13}C_{\text{biomass}}=\delta^{13}C_{\text{autotrophy}}+\delta^{13}C_{\text{heterotrophy}} \end{array}$$

(3) $$\begin{array}{l} \delta^{13}C_{\text{autotrophy}}=\left(\delta^{13}C-DIC-\varepsilon_{\text{autotrophy}}\right)\\ \times \;f_{\text{Microbial}-\text{DIC}} \end{array}$$

(4) $$\begin{array}{l} \delta^{13}C_{\text{heterotrophy}}=\left(\delta^{13}C-OC-\varepsilon_{\text{heterotrophy}}\right)\\ \times \;f_{\text{Microbial-DOC}/\text{OC}} \end{array}$$

Weighted fractionation factors used in the mixing models were based on the relative abundance of each CO_2_-fixing microorganism and the predicted autotrophic fractionation factor (ε-value) for each phylotype (Table 2); fractionation factors were estimated based on carboxylase sequence similarity with cultured relatives (Jahnke et al., 2001; House et al., 2003; Jennings et al., 2014). For modeling purposes, the heterotrophic fractionation of ^13^C-OC (i.e., DOC or landscape OC with an average ^13^C content of ~ −23%) into microbial biomass was assumed to be 0% (Boschker and Middelburg, 2002).

**Table 2:** **Relative abundance of primary autotrophs distributed across different geothermal habitats in Yellowstone National Park and predicted fractionation factors (ε) for the three major carbon dioxide fixation pathways observed in these populations**.

**CO_2_ Fixation Cycle/Primary Autotrophs^a^**	**Fract. Factor^b^**	**Sulfidic sediments^c^**						**Iron-oxide mats**				**Filam. streamers**			
		**CH**	**JC2_E**	**MG**	**CIS**	**JC3_A**	**WSH^e^**	**JC2_B^e^**	**BE_D**	**ECH_B**	**OSP_B**	**Drgn_B**	**NG**	**OCT_B**	**CON_B**
**r-TCA Cycle**	**ε (‰)**	**Relative Abundance^d^**													
*Hydrogenobaculum*	5.5		15.9						2.5	2.6	3.1	29.7			
*Sulfurihydrogenibium*	5.5						2						94.3		
*Thermocrinis* spp.	3.3				3.6	1.2								23.4	39.5
**3-HP/4-HB Cycle**	**ε (‰)**														
*Acidianus* spp.	0.2							10.8							
*Metallosphaera*	2.5							3	7.2	9.7	13.2				
*Sulfolobus* spp.	0.2		2.5						2.3	2.4	2.5				
Sulfolobales T1	3.1	68.9		1.6	9.4	4.2					0.1	6.1			
Sulfolobales T2	3.6	7.9			0.8			84.5	0.1	0.6	1.1				
**DC/4-HB Cycle**	**ε (‰)**														
Thermoproteales T1	2			11.8	26.2	19.5	0.1		0.2	4.7	11.8	6.6			
Thermoproteales T2	2.9			2	23.6	34.2	4.3							9.2	20.3

a *Candidate autotrophs established based on presence of CO_2_ fixation pathways in random metagenome sequence. Sulfolobales T1 and T2 = Sulfolobus and Stygiolobus-like, respectively. Thermoproteales T1 and T2 = Caldivirga/Vulcanisaeta and Thermoproteus/Pyrobaculum-like, respectively*.

b *Fractionation factors (ε, %) estimated based on cultured relatives*.

c *CH, Crater Hills; JC2_E, Joseph's Coat Spring 2; MG, Monarch Geyser; CIS, Cistern Spring; JC3_A, Joseph's Coat Spring 3; WSH, Washburn Hot Spring; DS, Dragon Spring; NG, Narrow Gauge; OCT, Octopus Spring; CON, Conch Spring; BE, Beowulf East; GRN, Grendel Spring; ECH, Echinus Geyser; OSP, 100 Spring Plain (see Table S1 for other details)*.

d *Relative abundance values are based on metagenome read recruitment to de novo phylotype genomes, unless otherwise noted*.

e *Relative abundance estimates based on iTag data*.

*Other community members are identified in Supplemental Information (Table S6)*.

## Results

### ^13^C content of DIC, DOC, and landscape OC

**Figure 1:**
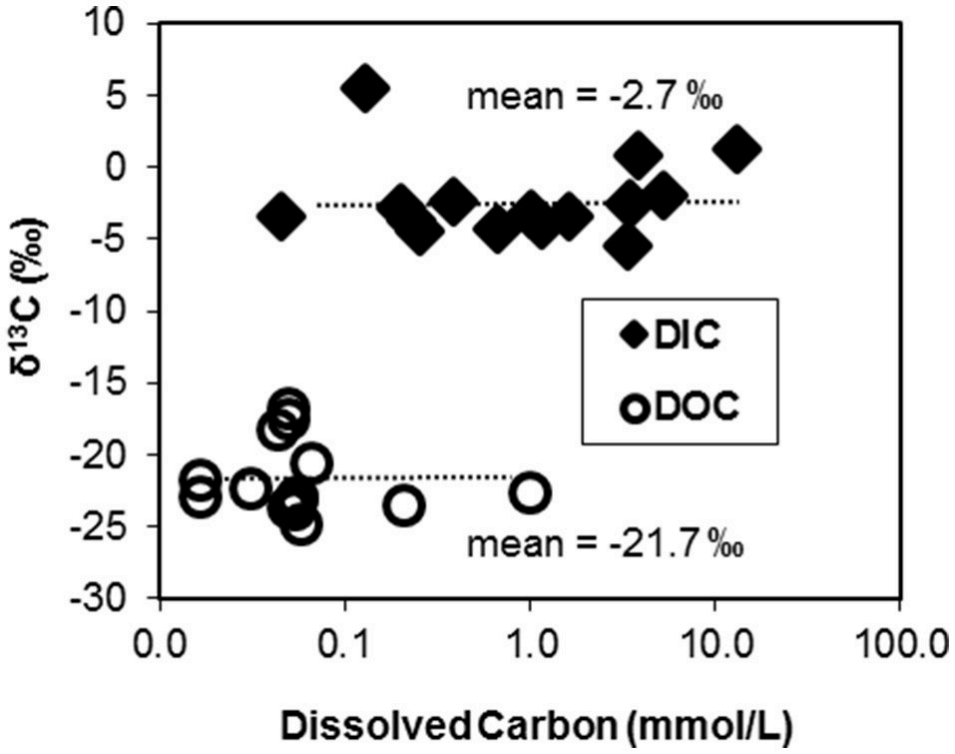
**Stable carbon isotope contents (δ^**13**^C, %) of dissolved organic C (DOC) and dissolved inorganic C (DIC) pools plotted as a function of the concentration of C in 15 geochemically diverse high-temperature environments of Yellowstone National Park, WY, USA**.

The ^13^C contents of DIC and DOC measured across 15 geothermal systems averaged −2.7% (± 2.8) and −21.7% (± 2.3), respectively (Figure 1). The ^13^C contents of each C “pool” were fairly well-constrained across geochemically diverse sites with a few exceptions. The larger ^13^C-DIC value at Crater Hills (5.4%) appeared to be the primary outlier relative to other diverse sites including Mammoth Hot Springs (MHS) where high DIC concentrations (up to 15–20 mM) reflect the dissolution of Madison limestone during ascent of hydrothermal fluids (Fouke, 2011). Most of the δ^13^C-DOC values from geothermal sites were close to the average δ^13^C value obtained for landscape organic C (−23.7%), although a few springs exhibited slightly heavier δ^13^C values near −17 to −18% (Table 1).

The majority of geothermal source waters and outflow channels sampled in this study exhibited DIC concentrations that were ~10-fold higher than DOC (Figure 1, Table 1). Most of the observed DOC levels were near the analytical detection limit (~20–40 μM C), which has also made it difficult to obtain compositional data of DOC in these systems. The high concentrations of DIC relative to DOC in these geothermal springs suggest that CO_2_ fixation may be an important source of biomass C in many high-temperature communities, and may provide reduced C compounds that are utilized by heterotrophic populations.

The ^13^C isotope content of potential landscape C sources was determined for plant needles, leaves, algal and cyanobacterial communities, animal dung (e.g., bison and wolf) and soils collected from areas near and/or upslope of nearly all geothermal springs sampled. The average δ^13^C values of landscape samples was −23.7% (*n* = 29) and ranged from −16 to −28.6% (Table S1), with the exception of *Zygogonium* spp. algal mat samples (i.e., δ^13^C = −10.7%), which were not found within the immediate upstream flow path for mats sampled in this study. The average ^13^C isotope content of leaf, soil, and dung samples (δ^13^C ~ −24%) reflects the importance of C_3_ photosynthesis as the primary C source in landscape samples (Ehleringer and Monson, 1993). Landscape OC may contribute to the total C present in geothermal samples either as C utilized to produce microbial biomass and/or as direct landscape inputs.

### ^13^C content of chemotrophic microbial communities

The ^13^C contents of all microbial community samples were between the average δ^13^C values for DIC and OC (DOC or landscape OC; Table 1). Nearly all microbial samples exhibited ^13^C values significantly higher (less negative) than ^13^C-DOC or ^13^C-OC from landscape sources, which provides direct evidence for the presence of DIC-derived microbial biomass. Several sulfur sediment communities (i.e., Monarch Geyser, Joseph's Coat Hot Springs [JC3_A, JC2_E]) exhibited ^13^C values ranging from ~−6 to −16%, and all Fe(III)-oxide sites exhibited ^13^C values ranging from ~−8 to −16%, which indicate significant fractions of DIC-derived biomass in these microbial communities. The ^13^C content of filamentous “streamer” samples (containing different members of the Aquificales) showed that these communities also exhibit large, although variable extents of CO_2_ fixation (Table 1). A more accurate determination of the amount of DIC-derived microbial C present in these communities is possible after considering the appropriate fractionation factors (below) for incorporation of ^13^C-DIC into microbial biomass, which is dependent on the primary microorganisms present and their respective pathways for CO_2_ fixation.

### Relative abundance of CO_2_-fixing microorganisms

Bacteria and/or archaea with evidence for known CO_2_ fixation pathways were identified in the microbial communities studied using random metagenome sequencing (Table 2). The relative amount(s) of autotrophic populations varied considerably across the habitat types evaluated, and ranged from as high as 94% at MHS (a streamer community dominated by *Sulfurihydrogenibium* spp.), to as low as ~6% in Washburn Spring (sediment community), which contains a diverse mixture of archaeal and bacterial heterotrophs (Inskeep et al., 2013b). Microbial community members not containing established CO_2_ fixation pathways were lumped into heterotrophic populations (Table S6) for purposes of interpreting ^13^C stable isotope data; the phylogeny and metabolic attributes of many of these populations are elucidated in more detail in other studies (Inskeep et al., 2013b; Beam et al., 2014, 2016a,b; Jay et al., 2014, 2015, 2016; Colman et al., 2016).

The most common phylotypes that contained CO_2_ fixation pathways across these habitats included different members of the Sulfolobales (pH < 4), the Thermoproteales (pH 3–9), and the Aquificales (pH 3–9; Table 2). *Metallosphaera yellowstonensis* and several *Sulfolobus*-like organisms present in low pH Fe(III)-oxide microbial mats and sulfur-sediments contained key genes of the 3-HP/4-HB pathway, and prior work has shown that *M. yellowstonensis* str. MK1 is a facultative autotroph in these habitats (Kozubal et al., 2008; Jennings et al., 2014). *Caldivirga, Thermoproteus*, and *Pyrobaculum* spp. were the predominant phylotypes with genomic evidence for CO_2_ fixation in higher pH (pH ~ 5–9) sulfur-sediment systems (Table 2) via the DC/4-HB pathway (Ramos-Vera et al., 2009; Jay et al., 2016). Pathway completeness of the DC/4-HB cycle in the Thermoproteales T1 and T2 phylotypes was difficult to assess based on lower coverage Sanger sequencing (Table S3); however, analysis of Illumina metagenomes from these same sites (Table S4) provided support for complete DC/4-HB CO_2_ fixation pathways in the Thermoproteales T2 (Pyrobaculum/Thermoproteus) phylotypes. This is consistent with genomic and/or physiologic data of cultured members of these genera (Schäfer et al., 1986; Ramos-Vera et al., 2009; Jay et al., 2015).

Members of the Aquificales were identified in acidic sulfur streamers and Fe(III)-oxide mats (i.e., *Hydrogenobaculum* spp.), circumneutral sulfidic streamers (*Sulfurihydrogenibium* spp.), and slightly-alkaline (pH ~ 8–9) streamer communities (*Thermocrinis* spp.; Table 2). All of the Aquificales phylotypes contained nearly complete pathways necessary for the fixation of CO_2_ via the reverse TCA cycle, although slight variations exist regarding the mechanism of citrate cleavage among the Aquificales lineages found in YNP (Takacs-Vesbach et al., 2013; Table S3).

Fractionation factors (ε) for the incorporation of CO_2_ into *Metallosphaera* biomass and other close Sulfolobales relatives via the 3-HP/4-HB pathway range from 0.2 to 3.6% (House et al., 2003; Jennings et al., 2014). For the DC/4-HB pathway, reported ε-values range from 2.0 to 2.9% in Thermoproteales isolates (House et al., 2003). Fractionation factors for Aquificales isolates range from 3.3 to 5.5%, based on investigation of *Hydrogenobaculum* and *Thermocrinis* spp. (Jahnke et al., 2001; House et al., 2003). Consequently, fractionation factors for the primary CO_2_ fixation pathways identified in the dominant autotrophic populations present in these communities range from ~0.2 to 5.5%.

### Extent of autotrophy

The extent of autotrophy cannot be ascertained directly from the relative abundance of populations with autotrophic potential (i.e., random metagenome data), in part because most of these organisms are not obligate CO_2_-fixers. These microorganisms are facultative autotrophs and may obtain a fraction of their C from organic carbon sources. Moreover, the presence of landscape OC in a microbial mat sample will result in less ^13^C, and must be accounted for as a possible contribution to sample ^13^C values. Consequently, the ^13^C values of mat, streamer or sediment samples reflect contributions from DIC-derived and OC-derived microbial C, as well as landscape OC (Equation 1); sample ^13^C values were predicted using mixing models with appropriate fractionation factors for incorporation of DIC or OC (i.e., ε = 0%).

**Figure 2:**
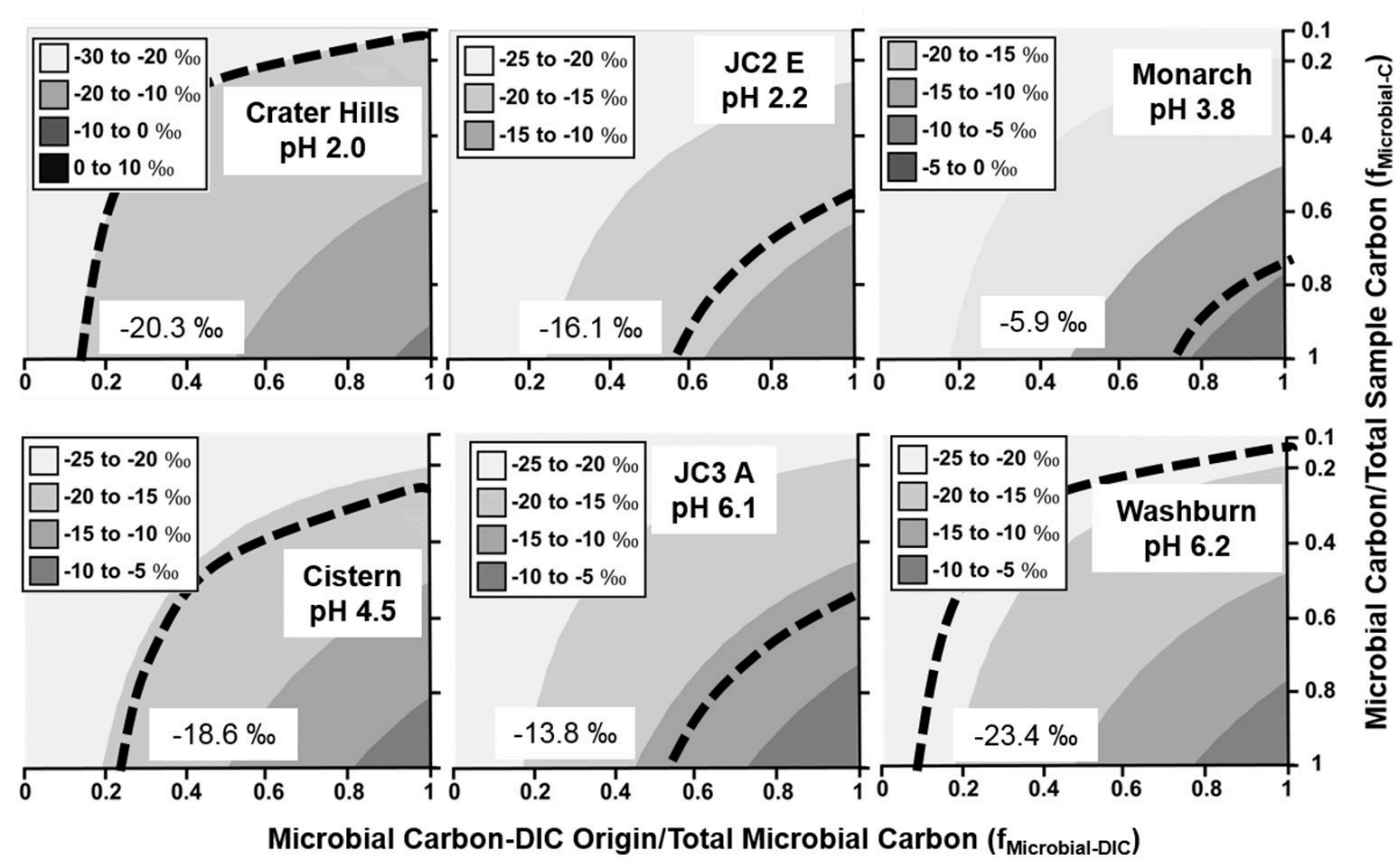
**Stable ^**13**^C isotope mixing models of sulfur-sediment microbial communities**. The fraction of microbial C of DIC origin relative to total microbial C (f_Microbial-DIC_) (x-axis) and the fraction of microbial C to total sample C (f_Microbial-C_) (y-axis) were used to model (Equation 1) the distribution of possible sample ^13^C contents (gray scale). Dotted lines indicate possible combinations of f_Microbial-DIC_ and f_Microbial-C_ that are consistent with the observed ^13^C values (‰) of microbial samples (boxed).

**Figure 3:**
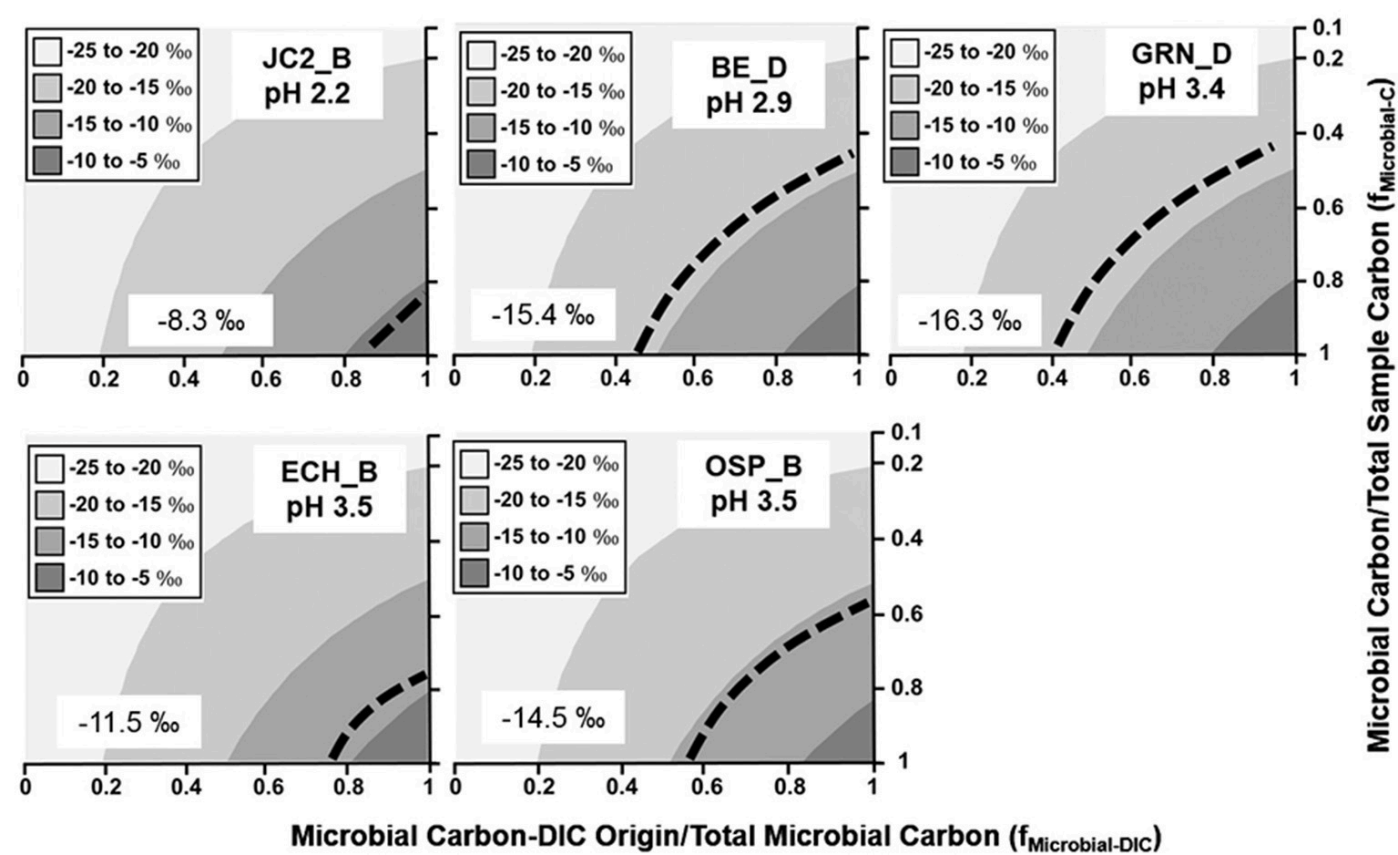
**Stable ^**13**^C isotope mixing models of acidic Fe(III)-oxide microbial communities**. The fraction of microbial C of DIC origin relative to total microbial C (f_Microbial-DIC_) (x-axis) and the fraction of microbial C to total sample C (f_Microbial-C_) (y-axis) were used to model (Equation 1) the distribution of possible sample ^13^C contents (gray scale). Dotted lines indicate possible combinations of f_Microbial-DIC_ and f_Microbial-C_ that are consistent with the observed ^13^C values (‰) of microbial samples (boxed).

**Figure 4:**
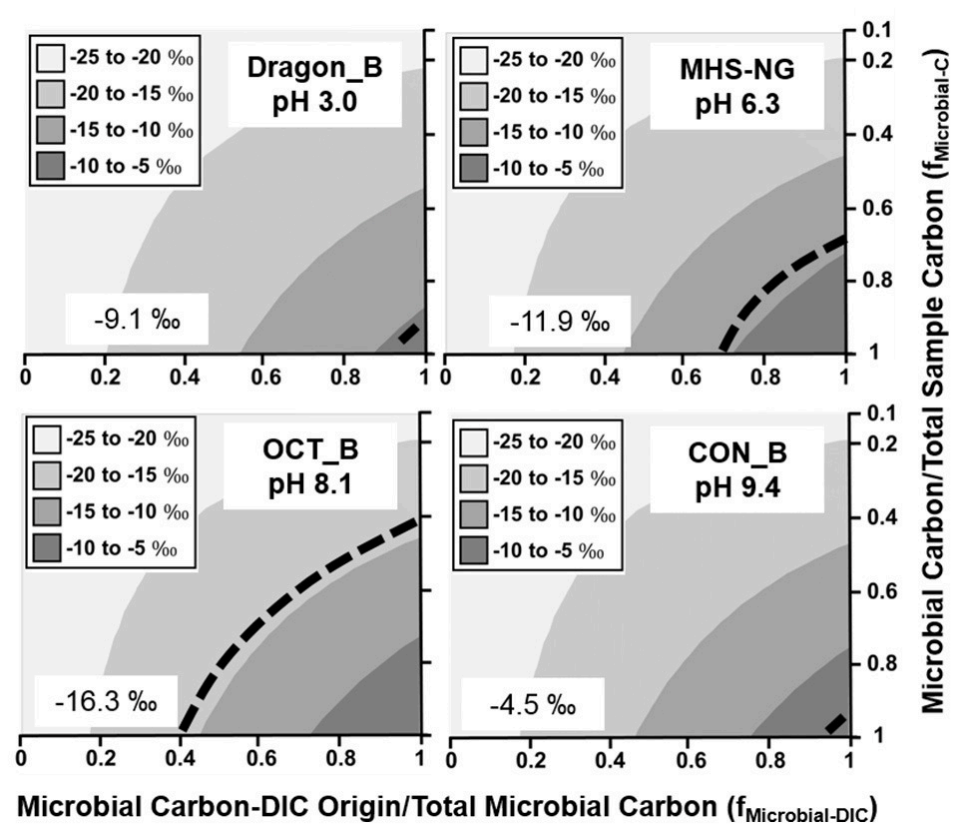
**Stable ^**13**^C isotope mixing models of thermophilic filamentous “streamer” communities**. The fraction of microbial C of DIC origin relative to total microbial C (f_Microbial-DIC_) (x-axis) and the fraction of microbial C to total sample C (f_Microbial-C_) (y-axis) were used to model (Equation 1) the distribution of possible sample ^13^C contents (gray scale). Dotted lines indicate possible combinations of f_Microbial-DIC_ and f_Microbial-C_ that are consistent with the observed ^13^C values (‰) of microbial samples (boxed).

Weighted average fractionation factors (ε) based on the relative abundances of autotrophic populations (Table 2) were used as input values for predicting the sample ^13^C contents as a function of DIC-derived microbial C relative to total microbial C, and the ratio of microbial C to total mat C (Equation 1). Although the amount of microbial C relative to total mat C is difficult to determine accurately, the measured ^13^C contents constrain estimates of this ratio as a function of the fraction of DIC-derived microbial C to total microbial C (Figures 2–**4**). Due to the small range of fractionation factors for conversion of ^13^C-DIC into microbial biomass compared to the large differences in δ^13^C-DIC and δ^13^C-OC, the isotope mixing models were relatively insensitive to the weighted average fractionation factors obtained from the relative abundance of specific autotrophs.

With the exception of high-OC sediments in Crater Hills and Washburn Spring (Figure 2), sulfur-sediment communities exhibited significant incorporation of DIC across a wide pH range from 2.0 to 6.2 (Figure 2). For example, the minimum fractions of DIC-microbial C in sulfur-sediments from Joseph's Coat (JC2_E and JC3_A) and Monarch Geyser were >50%. The primary autotrophs in low pH (pH < 3.0) sulfur systems include members of the Sulfolobales and Aquificales (*Hydrogenobaculum* spp.), and members of the Thermoproteales (i.e., *Thermoproteus* and *Pyrobaculum*-like organisms) across a pH range from ~4 to 9 (Table 2). Integration of ^13^C data and metagenome analysis show that these populations are responsible for the fixation of inorganic C *in situ*.

The lower fraction of DIC-microbial C relative to total microbial C (x-axis, Figure 2) observed in Crater Hills and Washburn Springs suggests contributions from heterotrophy (incorporation of OC) and/or landscape OC in these samples. Crater Hills is inhabited primarily by Sulfolobales populations, which are capable of CO_2_ fixation via the 3-HP/4-HB pathway (Table 2), yet the minimum fraction of DIC-microbial C to total microbial C is quite low (< ~20%). Thus, the Sulfolobales populations (2 predominant phylotypes) at Crater Hills may utilize significant amounts of OC, and/or the sample contains a high fraction of non-microbial C from landscape sources. For example, the extent of autotrophy at Crater Hills could exceed 50% of the microbial C if landscape OC were to have contributed >80% of the total sample C (Figure 2, top left). In contrast, Washburn Spring sediments (pH 6.2) contained a much lower relative abundance of autotrophic microorganisms (Table 2). The low ^13^C contents may reflect high fractions of heterotrophic consumption of OC and/or high fractions of non-microbial C from landscape sources. The Washburn Spring and Crater Hills sites are both located in landscape depressions and receive significant amounts of vegetative debris and surface runoff, which contributes to the large fraction of ^13^C-OC in these sediments.

The minimum fraction of DIC-microbial C in acidic Fe(III)-oxide mats ranged from near 40–85% (Figure 3), and was consistent across several different types of Fe(III)-oxide systems (Kozubal et al., 2012). Moderately acidic (pH ~ 3.0–3.5), poorly-crystalline, high-arsenic Fe(III)-oxide mats in Norris Geyser Basin (OSP_B, BE_D) were comprised of 40–60% DIC-microbial C. An additional Fe(III)-oxide mat containing less arsenic (Kozubal et al., 2012) also showed significant fractions of DIC-microbial C (ECH_B). Moreover, an extremely mineralized Fe(III)-oxide mat (Kozubal et al., 2012) with crystalline goethite and hematite (JC2_B, pH 2.2) contained a large fraction of organisms capable of CO_2_ fixation (primarily Sulfolobales), and a minimum of 85% of the microbial C was predicted to be of DIC-origin (Figure 3). Consequently, definitive evidence for significant extents of CO_2_ fixation was obtained for numerous Fe(III)-oxide communities, and in all cases, metagenome sequence analysis confirmed the importance of *Hydrogenobaculum* spp. (reverse TCA cycle) and members of the Sulfolobales (e.g., *M. yellowstonensis* and *Stygiolobus*-like spp.; 3-HP/4-HB cycle) as the primary autotrophic organisms in these systems, despite differences in pH (~2–4) and Fe(III)-oxide mineralogy (Inskeep et al., 2004; Kozubal et al., 2012).

Filamentous “streamer” communities were sampled across a wide range of geochemical conditions (pH 3.0–9.4; high H_2_S *vs*. high O_2_), and in all cases, a significant fraction of DIC-microbial C was observed (Figure 4). Different streamer communities included the low-pH systems dominated by *Hydrogenobaculum* spp. (Dragon Spring), the circumneutral (pH 6.3) springs inhabited by *Sulfurihydrogenibium* spp. (Mammoth Hot Springs) and the higher pH (8–9) systems (Octopus and Conch Springs) that contain significant fractions of *Thermocrinis* spp. (Table 2). The fixation of inorganic C by these autotrophs supports a diverse group of different heterotrophic organisms (Table S6) distributed across this habitat range (Takacs-Vesbach et al., 2013; Colman et al., 2016), and demonstrates the importance of the Aquificales and the r-TCA cycle (Table 2, Table S3) in establishing high-temperature filamentous “streamer” communities.

## Discussion

The fixation of CO_2_ provides reduced biomass C to a wide range of heterotrophic assemblages (Table S6) that comprise each of the microbial communities studied. The primary thermophilic bacteria and archaea with known pathways for CO_2_ fixation, and which were present in sites with significant fractions of DIC-derived microbial C included representatives of the Aquificales (three different genera), as well as the Sulfolobales and Thermoproteales (phylum Crenarchaeota). These chemolithoautotrophs obtain energy for CO_2_ fixation from the oxidation of reduced species including H_2_, H_2_S, S^0^, As(III), and Fe(II), and are distributed in large part based on their ability to utilize specific electron donor-acceptor combinations. For example, the respiration of autotrophic Sulfolobales and Aquificales populations is coupled with the reduction of O_2_ (e.g., Inskeep et al., 2013b; Takacs-Vesbach et al., 2013). However, several sub-oxic autotrophic members of the Thermoproteales likely reduce elemental S and/or arsenate (Jay et al., 2015, 2016).

Metagenome and stable isotope analyses were used to show that CO_2_ fixation occurs across extremely diverse geochemical conditions, including three predominant chemotrophic habitat types in YNP. Inorganic C fixation thus supports both autotrophic and heterotrophic populations in high-temperature communities. The autotroph-heterotroph associations implicated from these ^13^C stable-isotope measurements, combined with molecular data on community composition and function reveal that three major CO_2_ fixation pathways are responsible for supporting a diverse array of different heterotrophic populations that are adapted to different geochemical conditions (e.g., pH, oxygen, sulfur). The importance of CO_2_ fixation in thermal systems also suggests that trophic structure and autotroph-heterotroph interactions are consistent themes in microbial communities often thought to be analogs of environments supporting early life on Earth (Nisbet and Sleep, 2001).

Stable ^13^C isotope mixing models were used to constrain possible solutions to the observed ^13^C content of Fe(III)-oxide, sulfur-sediment and filamentous “streamer” samples. Results demonstrated that, at a minimum, 40–50% of the total microbial C in many systems analyzed was derived from the fixation of DIC (x-axis, Figures 2–4). Further resolution of the fraction of non-microbial C (i.e., landscape OC; y-axis, Figures 2–4) would assist in clarifying the extent of autotrophy, and this would be most beneficial in sediment environments of geothermal springs located in landscape depressions, which contain large fractions of OC-derived microbial biomass and/or significant inputs of OC from landscape sources. Geothermal sites in concave topography (e.g., Crater Hills, Washburn Springs) receive significant inputs of landscape OC through erosion, litter-fall and/or storm-water relative to convex systems (e.g., MHS, Conch Spring). Consequently, as it relates to the input of landscape carbon, topographic position is likely an important selective force in the establishment, assembly, and resulting composition of geothermal microbial communities.

Chemoorganoheterotrophs in these high-temperature communities obtain a fraction of biomass C from autotrophic community members (i.e., DIC-derived microbial C), in addition to DOC and/or landscape OC. The similar ^13^C contents of landscape OC and geothermal DOC preclude the separation of these possible OC sources to microbial community members. Although little is known regarding the composition and availability of DOC in geothermal habitats (and should be addressed in future studies), the observed concentrations of DOC are considerably lower than DIC in the majority of systems examined (Figure 1). Recent succession studies in Fe(III)-oxide microbial mats (Macur et al., 2004; Beam et al., 2016b) show that autotrophic organisms are early colonizers followed by increases in heterotrophs at later successional stages. These observations are consistent with the large fractions of DIC-derived microbial C observed in numerous Fe(III)-oxide mat systems (Figure 3). Higher concentrations of DOC were observed in Washburn Spring (1.0 mM, Table 1), and may explain the lower abundance of candidate autotrophs (~4%, Table 2) in sediments present at this site. Moreover, a significant fraction of the total microbial C in these sediments is not of DIC-origin (Figure 2). The low-pH elemental sulfur system at Crater Hills also contained a significant amount of DOC (~0.2 mM), and this may contribute to the lower apparent DIC contribution to microbial C at this site.

High-temperature communities across a wide range of geochemical conditions (e.g., sulfur sediments, Fe(III)-oxide mats, filamentous streamers) exhibited large fractions of DIC-derived microbial C. Although autotrophic organisms were present in all sites that exhibited large fractions of DIC-derived microbial C, the abundance of primary autotrophs in these systems does not necessarily correlate with the minimum fractions of DIC-derived C established using ^13^C mixing models. For example, the relative abundance of autotrophic microorganisms in sites exhibiting >90% DIC-derived C ranged from ~17 to 93% (Table 2). Consequently, other processes should be considered when interpreting the extent of DIC-derived microbial C vs. the relative abundance of primary autotrophs. Specifically, the relative growth and/or turnover rates of autotrophic vs. heterotrophic microorganisms may not be consistent with relative abundances based on DNA sequence analysis. Viral predation likely impacts community members differentially, and could be one of the more important factors explaining lower autotroph relative abundances in communities with large fractions of DIC-derived microbial C (i.e., >50%). Future work on the composition and microbial utilization of OC sources, the relative turnover rates of autotrophs vs. heterotrophs, and the activity of different community members (e.g., transcriptomics) will assist in defining the causal factors responsible for C cycling across these geochemically diverse systems.

## Author contributions

RJ: Conceived and co-designed experiments. Conducted bioinformatic analysis of CO_2_ fixation pathways. Analyzed carbon concentration and isotope data. Drafted and edited manuscript at all stages. JM, ZJ, JB, LW, MK, HK: Data collection and analysis. WI: Conceived and designed experiments. Data collection and analysis. Manuscript drafting and editing at all stages.

### Conflict of interest statement

The authors declare that the research was conducted in the absence of any commercial or financial relationships that could be construed as a potential conflict of interest.
