# Supplementary figures and images for: Integration of Metagenomic and Stable Carbon Isotope Evidence Reveals the Extent and Mechanisms of Carbon Dioxide Fixation in High-Temperature Microbial Communities
Source: Front Microbiol. 2017 Feb 3;8:88. doi: 10.3389/fmicb.2017.00088 (PMC5289995; doi:10.3389/fmicb.2017.00088)

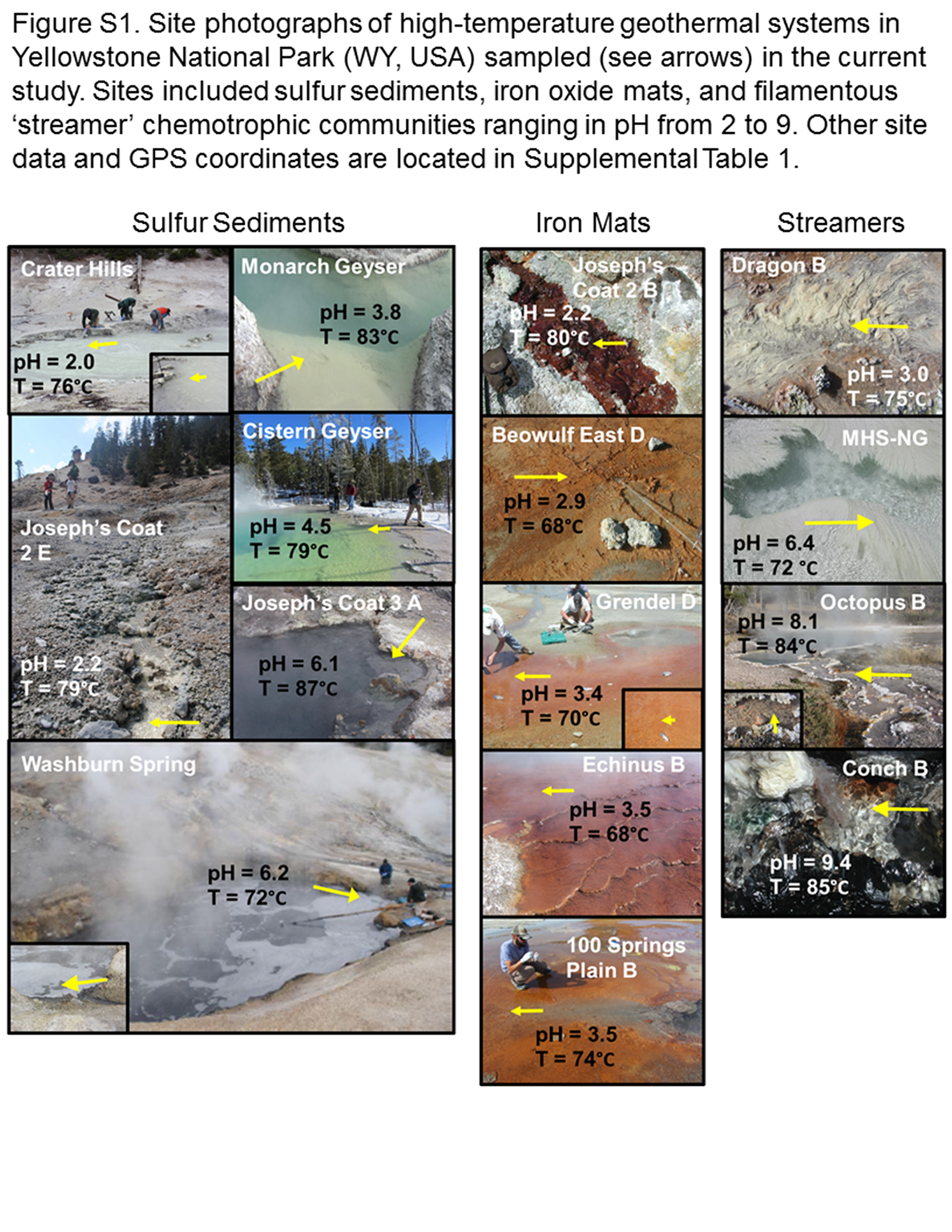

Supplement: Supplementary file 7 [file Image1.TIF]
